# Biochemical Characterization of Human Anti-Hepatitis B Monoclonal Antibody Produced in the Microalgae *Phaeodactylum tricornutum*

**DOI:** 10.1371/journal.pone.0139282

**Published:** 2015-10-05

**Authors:** Gaëtan Vanier, Franziska Hempel, Philippe Chan, Michael Rodamer, David Vaudry, Uwe G. Maier, Patrice Lerouge, Muriel Bardor

**Affiliations:** 1 Laboratoire Glycobiologie et Matrice Extracellulaire végétale Equipe d’Accueil 4358, Faculté des sciences et techniques, Université de Rouen, Normandie Université, Institut de Recherche et d’Innovation Biomédicale, Végétale Agronomie Sol Innovation, Mont-Saint-Aignan, France; 2 LOEWE Center for Synthetic Microbiology, Philipps-Universität Marburg, Marburg, Germany; 3 PISSARO Proteomic Platform, Normandie Université, Institut de Recherche et d’Innovation Biomédicale, Mont-Saint-Aignan, France; 4 Agilent Technologies, Waldbronn, Germany; 5 Institut Universitaire de France, Paris, France; New York State Dept. Health, UNITED STATES

## Abstract

Monoclonal antibodies (mAbs) represent actually the major class of biopharmaceuticals. They are produced recombinantly using living cells as biofactories. Among the different expression systems currently available, microalgae represent an emerging alternative which displays several biotechnological advantages. Indeed, microalgae are classified as generally recognized as safe organisms and can be grown easily in bioreactors with high growth rates similarly to CHO cells. Moreover, microalgae exhibit a phototrophic lifestyle involving low production costs as protein expression is fueled by photosynthesis. However, questions remain to be solved before any industrial production of algae-made biopharmaceuticals. Among them, protein heterogeneity as well as protein post-translational modifications need to be evaluated. Especially, *N*-glycosylation acquired by the secreted recombinant proteins is of major concern since most of the biopharmaceuticals including mAbs are *N-*glycosylated and it is well recognized that glycosylation represent one of their critical quality attribute. In this paper, we assess the quality of the first recombinant algae-made mAbs produced in the diatom, *Phaeodactylum tricornutum*. We are focusing on the characterization of their C- and N-terminal extremities, their signal peptide cleavage and their post-translational modifications including *N-*glycosylation macro- and microheterogeneity. This study brings understanding on diatom cellular biology, especially secretion and intracellular trafficking of proteins. Overall, it reinforces the positioning of *P*. *tricornutum* as an emerging host for the production of biopharmaceuticals and prove that *P*. *tricornutum* is suitable for producing recombinant proteins bearing high mannose-type *N*-glycans.

## Introduction

The biopharmaceuticals market is valued at greater than US$100 billion, which includes more than 200 product types [1]. Among the various categories (e.g., hormones, cytokines, and growth factors), monoclonal antibodies (mAbs) and their derivatives form the largest group of biopharmaceuticals. For example, in 2010 in the US, mAbs represented 36% of all protein therapeutics sold, with overall sales of US$18.5 billion [2]. In 2011, the global market for mAbs was estimated at $44.6 billion and forecast to rise at an annual growth rate of 5.3% to nearly $58 billion in 2016 (www.bccresearch.com/market-research/biotechnology). mAbs are actually used to prevent or treat a variety of serious medical conditions affecting millions of people annually. This includes diseases such as cancers, immune disorders, inflammation, and infectious diseases [3]. Today, Chinese Hamster Ovary (CHO) cell lines are considered as the gold-standard for the biopharmaceutical industry, due to their ability to carry out complex post-translational modifications, including human-like glycosylation [4]. CHO cell lines are currently responsible for the production of nearly 50% of the 28 mAbs marketed in the United States or European Union [5]. However, the constantly increasing need for large quantities of biopharmaceuticals, their high production cost in CHO cells, and factors related to virus contamination have encouraged the development of new alternative production systems. Among those, there is an increasing interest for the use of microalgae as bioreactors for large-scale production of biopharmaceuticals [6,7]. Similarly to CHO cells, microalgae combine high growth rates, easy handling, and the capacity to perform post-translational modifications such as *N-*glycosylation [8–11], which are crucial for biopharmaceutical folding, half-life and activity [12]. Moreover, the use of microalgae as cell factories, because of their phototrophic lifestyle, will allow reduction of the biopharmaceutical production costs [6,13]. Proofs-of-concept for biopharmaceutical productions in microalgae have been made [6,7]. Most of these productions have been performed in the green microalga, *Chlamydomonas reinhardtii* [14–17]. This includes successful production of the single-chain antibody directed against the glycoprotein D of the herpes simplex virus [14], human erythropoietin (EPO; [15]), vascular endothelial growth factor (VEGF; [16]) and the mAb 83K7C [17]. However, except for the recombinant EPO that was produced through genome nuclear expression [15], all other microalgae biopharmaceuticals have been produced through chloroplastic expression, which is unsuitable for protein post-translational modifications.

Recently, the diatom *P*. *tricornutum* has also been used to produce biopharmaceuticals. Indeed, successful production of monoclonal human antibodies directed against the Hepatitis B virus surface antigen (HBsAg), either secreted or retained in the endoplasmic reticulum (ER) has been reported [18, 19]. These algae-made recombinant antibodies were shown to be fully-assembled [18, 19] and functional *in vitro* [18].

Here, we biochemically characterize the HBsAg antibodies produced in *P*. *tricornutum* by analyzing their post-translational modifications including their *N-*glycosylation heterogeneity and their susceptibility to proteolytic degradation. A specific attention has also been paid to the signal peptide cleavage site. This represents a first attempt to assess the critical quality attributes of algae-made antibodies. Overall, this study brings new insights in regards to secretion, post-translational modification, and intracellular trafficking of proteins in diatoms. This will help with maximizing the potential of microalgae as a biopharmaceuticals production system.

## Materials and Methods

### Antibody production and purification

*P*. *tricornutum* cell lines producing human IgG antibodies against the Hepatitis B Virus surface protein (HBsAg, clones CL4mAb+DDEL #12 and CL4mAb-DDEL #11, [18] and [19]) were grown under agitation (150 rpm) and continuous illumination (80 μmol photons per m^2^ per sec) in f/2 medium containing 1.5 mM NH_4_Cl as nitrogen source. At a density of approximately 7 x 10^6^ cells.ml^−1^ of culture were shifted to fresh f/2 medium containing 0.9 mM NaNO_3_ instead of NH_4_Cl to induce antibody expression for two days. Subsequently, intracellular antibodies from the cell extract of strain CL4mAb+DDEL #12 were purified through affinity chromatography using Protein A sepharose according to [18]. Secreted antibodies from the culture medium of cell line CL4mAb-DDEL #11 were concentrated with centrifugal filter columns (cut off 10 kDa) as described in [19] and then resuspended in water. For antibody quantification, the Easy-Titer Human IgG assay kit was used according to the manufacturer instructions. All antibody samples were frozen in liquid nitrogen and stored at -80°C before proceeding with further biochemical analyses. All the experiments described below have been repeated independently at least twice.

### SDS-PAGE analysis

The SDS-PAGE gel run in non-reducing conditions has been performed and stained according to [18, 19].

### NuPAGE Bis-Tris Gel electrophoresis

Protein marker (5 μL of PageRuler Plus Prestained Protein Ladder, BP3603 Series, Thermo Scientific) and purified recombinant antibodies (3 to 30 μg diluted in 1X NuPAGE LDS Sample Buffer [50 mM Tris-HCl pH 6.8, 2% SDS, 6% glycerol, 1% β-mercaptoethanol, 0.004% bromophenol blue]) were loaded and separated on a NuPAGE Bis-Tris Gel 4–12%, 10 wells (Life Technologies) using reducing conditions. Separation was performed at a constant voltage 180V in the NuPAGE MOPS SDS running buffer (Life Scientific). After the migration, the gel was stained with Coomassie Brilliant Blue R–250 (Thermo Scientific).

### Quantification of the site occupancy by densitometry

Evaluation of the *N*-glycosylation site occupancy percentage was assessed by densitometry using the ImageJ software. Briefly, for each interesting lane of the NuPAGE Bis-Tris gel electrophoresis, squares were used to select the zone of interest. Then, the selection was treated using the submenu of ImageJ software with the option called gel analysis thus generating lane profile plots. Lines were drawn manually on the plots to define precisely each area. Finally, the peak areas under the curve were measure using the wand tool.

### Sample preparation before mass spectrometry analysis

The heavy and light chains of the antibodies (30 μg) were separated on a NuPAGE Bis-Tris gel electrophoresis as described earlier. The two bands corresponded respectively to the heavy and light chains of the antibody were excised from the gel and cut into several pieces. Then, the gel pieces were washed several times with a solution mixture composed of 0.1M NH_4_HCO_3_ pH 8 and 100% CH_3_CN (v: v). The samples were dried down in a SpeedVac centrifuge (Thermo Fisher) for few minutes. After a reduction step with 0,1M dithiothreitol (DTT) for 45 min at 56°C and alkylation with 55 mM iodoacetamide (IAA) for 30 min at room temperature in the dark, proteomic-grade trypsin was added (1μg per protein band; Promega) and placed at 4°C during 45 min prior to an overnight incubation at 37°C. After the protease digestion, the gel pieces were incubated subsequently in a 50% CH_3_CN solution, 5% formic acid solution, 0,1M NH_4_HCO_3_, 100% CH_3_CN and finally in 5% formic acid to extract the resulting peptide and glycopeptide mixture. The sample was finally dried down before further analysis using the high sensitivity nanospray LC-Chip MS QTOF from Agilent Technologies, U.S.A.

### Protein identification and site-specific distribution of *N*-glycans

MS analyses were performed using the nano-LC1200 system coupled to a QTOF 6520 mass spectrometer equipped with a nanospray source and a LC-Chip Cube interface (Agilent Technologies). Briefly, peptide and glycopeptide mixture was enriched and desalted on a 360 nL RP-C18 trap column and separated on a Polaris (3-*μ*m particle size) C18 column (150 mm long x 75 *μ*m inner diameter; Agilent Technologies). A 33-min linear gradient (3–75% acetonitrile in 0.1% formic acid) at a flow rate of 320 nL.min^−1^ was used, and separated peptides were analyzed with the QTOF mass spectrometer. Full autoMS scans from 290 to 1700 *m/z* and autoMS/MS from 59 to 1700 *m/z* were recorded. In every cycle, a maximum of 5 precursors sorted by charge state (2^+^ preferred and single-charged ions excluded) were isolated and fragmented in the collision cell. Collision cell energy was automatically adjusted depending on the *m/z*. Scan speed raise based on precursor abundance (target 25000 counts/spectrum) and precursors sorted only by abundance. Active exclusion of these precursors was enabled after 3 spectra within 1.5 min, and the threshold for precursor selection was set to 1000 counts.

### Peptide N-glycosidase F digestion

The peptide *N-*glycosidase F (PNGase F; Roche) digestion was performed on *P*. *tricornutum* peptides and glycopeptides mixture according to [8].

### Interpretations of the MS spectra

Raw data were analyzed using MassHunter (B.06.00; Agilent Technologies). First, compound list was extracted using the “*Find Compounds by Molecular Features*” submenu of Masshunter Bioconfirm workflow (Agilent Technologies) with the following parameters: peaks filter with height, 1000 counts; peak spacing tolerance, 0,0025 m/z, plus 7,0 ppm; charge state, 3 maximum; isotope model, peptides. Then, using the “*Define and Match sequences*” submenu, heavy and light chains were imported and were edited with cysteine carbamidomethylation and methionine oxidation as global modifications and with trypsin digestion including 2 miss cleavages maximum. The sequences were matched with the extracted compound lists. The corresponding proposed spectral annotations were checked manually and the sequence coverage was calculated manually. Moreover, the extracted ion chromatogram (EIC) was selected to interpret manually glycans, glycopeptides and tryptic peptides MS and MS/MS spectra.

The LC-MS and MS-MS data have been uploaded to the Figshare public repository and are accessible through the link: http://figshare.com/articles/Production_of_glycosylated_human_anti_Hepatitis_B_monoclonal_antibodies_in_the_microalgae_Phaeodactylum_tricornutum/1489703.

### SignalP 4.1 prediction server

SignalP 4.1 server (http://www.cbs.dtu.dk/services/SignalP/) was used to predict the presence and location of the signal peptide cleavage sites in the protein sequences. The input sequences (mAb heavy and light chains) in FASTA format were used. For each chain, submission was performed choosing eukaryotes as organism group, D-cutoff values by default, PNG for graphics output and a standard output format.

### Relative quantification of glycoforms

The peak intensities of the tri- and dicharged ions were added for each individual glycopeptide and the resulting value was used to calculate the relative percentage of each glycoform which was normalized to the sum of peak intensities of all the glycoforms.

## Results and Discussion

### Assembly of full-length recombinant monoclonal antibodies

**Fig 1:**
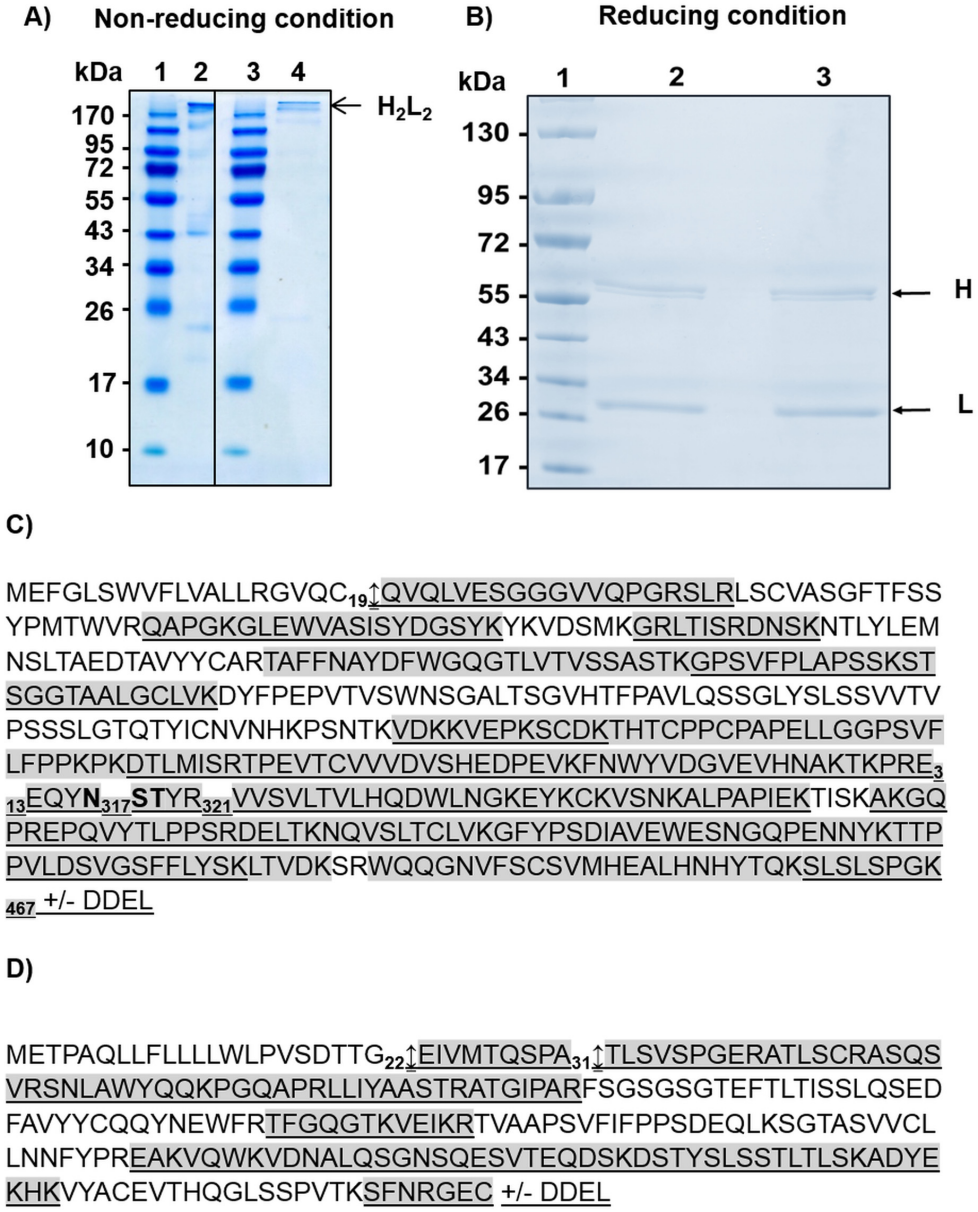
Protein analysis of algae-made HBsAg recombinant antibodies. **A)** SDS-PAGE gel in non-reducing conditions and stained with Coomassie blue. Lane 1: Molecular Weight; Lane 2: secreted mAb (1 μg); Lane 3: Molecular Weight; Lane 4: ER-retained mAb (1 μg). **B)** NuPAGE gel electrophoresis of mAbs produced in *P*. *tricornutum*. The gel has been run under reducing conditions and was stained with Coomassie blue. Lane 1: Molecular Weight; Lane 2: secreted mAb (3 μg); Lane 3: ER-retained mAb (3 μg). **C)** Heavy chain protein sequence of the antibody directed against HBsAg produced in *P*. *tricornutum* (GenBank accession number JF970210) plus or minus the DDEL ER-retention signal. This sequence include the signal peptide and the methionine is the first amino acid. **D)** Light chain protein sequence of the antibody directed against HBsAg produced in *P*. *tricornutum* (GenBank accession number JF970211) plus or minus the DDEL ER-retention signal. This sequence include the signal peptide and the methionine is the first amino acid. The peptides highlighted in grey are the ones which have been observed by LC-ESI MS analysis of tryptic digests for the heavy chain of the secreted mAb and for light chains of both ER-retained and secreted mAbs. The underlined peptides are the ones which have been observed by LC-ESI MS analysis of tryptic digests for the heavy chain of the ER-retained mAb. The heavy chain potential *N-*glycosylation site is in bold (N_317_ST). The arrows indicate the signal peptide cleavage sites.

Monoclonal antibodies (mAbs) directed against the Hepatitis B virus Antigen (HBsAg) have been produced recombinantly in the diatom, *P*. *tricornutum*, as previously described [18,19]. Those mAbs were either produced with (ER-retained mAb) or without (secreted mAb) a C-terminal DDEL (Asp-Asp-Glu-Leu) ER-retention signal. The ER-retained mAb was extracted and purified from the cell extracts using affinity chromatography with Protein A-sepharose according to [18]. The secreted mAb was concentrated from the culture medium using centrifugal filter columns as described in [19]. To analyze the full-length assembly of the two mAbs, the samples were separated by gel electrophoresis in non-reducing conditions (Fig 1A). A band H_2_L_2_ has been observed demonstrating that *Phaeodactylum tricornutum* is able to produce fully-assembled mAbs. However, other fragments could also be detected (Fig 1A). Those could correspond to misfolded or incorrectly assembled proteins or degradation fragments. A quantification of these products was performed directly on the stained gel using a densitometry analysis. In the retained version of the recombinant antibody, the fully-assembled antibody represents around 70% of the protein while in the secreted version, it represents up to 74% (Fig 1A).

### Protein sequence analysis of recombinant monoclonal antibodies

To assess the quality of, and biochemically characterize, the mAbs, a glycoproteomic approach using Agilent Technologies LC-Chip Cube MS with a high resolution QTOF mass spectrometer was performed. The heavy and light chains of the algae-made antibodies were first separated on a NuPAGE Bis-Tris gel *via* electrophoresis. This allowed detection of two major bands at the expected molecular weights for antibody heavy and light chains (Fig 1B). These bands were then excised from the gel, alkylated with iodoacetamide, and finally digested with trypsin. The resulting tryptic peptides were analyzed on a nano-LC1200 system coupled to a QTOF 6520 mass spectrometer. Peptide coverages of 55% for the heavy chain with DDEL and 70% for the heavy chain without DDEL were obtained (Fig 1C). With regards to light chains, 60% of tryptic peptides were identified by LC-ESI MS in both cases (Fig 1D). Large size tryptic digest that are not ionized efficiently by mass spectrometry explained the overall 55 to 70% peptide coverage.

### C-terminal ends of algae-made mAbs

**Fig 2:**
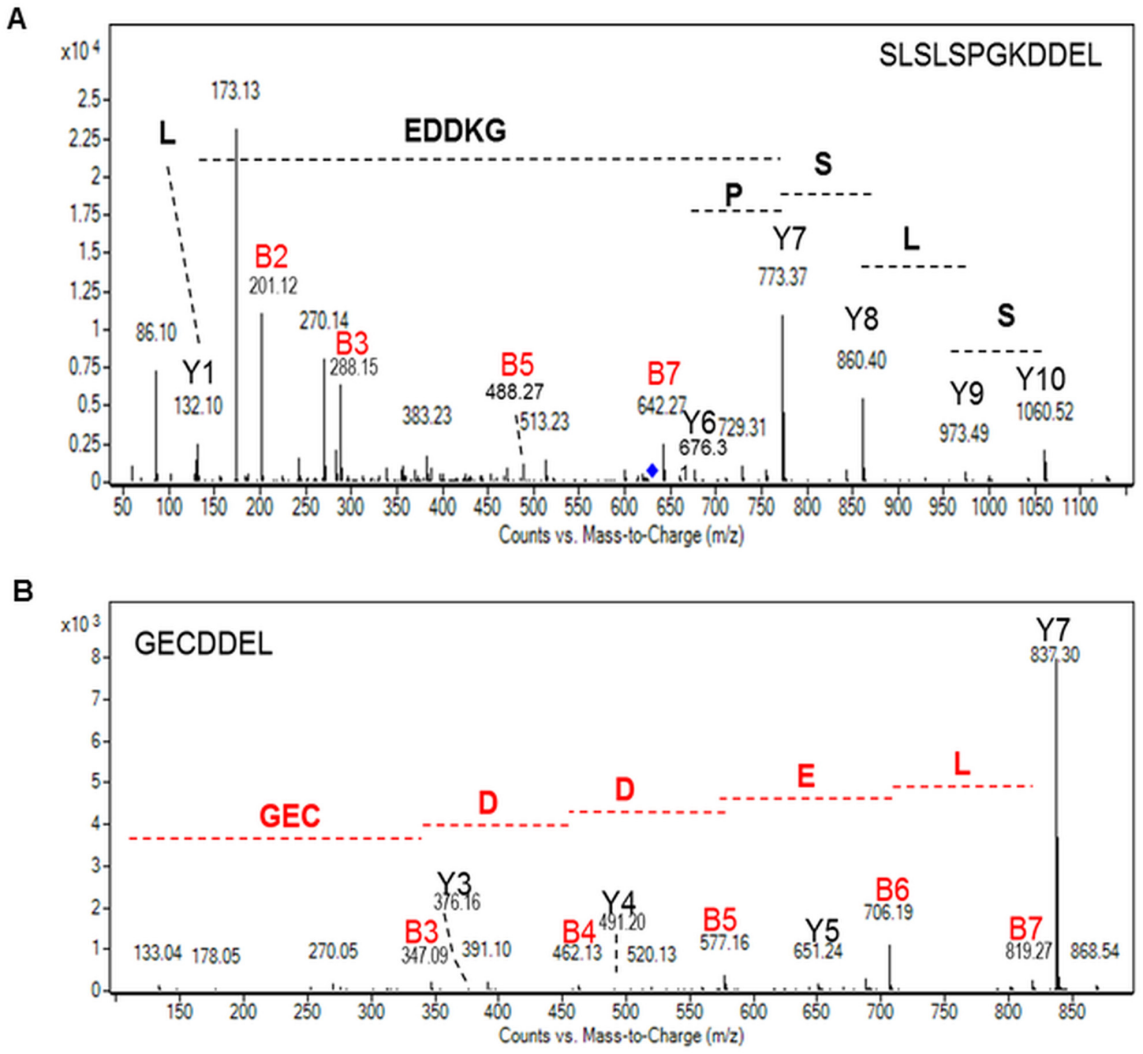
Mass spectrometry analysis of the C-terminal peptides of the heavy and light chains of the ER-retained mAb produced in *P*. *tricornutum*. **A)** MS/MS spectrum of the S_460_LSLSPGKDDEL_471_ ([M+2H]^2+^ precursor ion at *m/z* 630,82). **B)** MS/MS spectrum of the G_234_ECDDEL_240_ ([M+H]^+^ precursor ion at *m/z* 837, 29). For both spectra, Y and B ions have been identified and the peptide sequence can be rebuilt from either ones.

To ensure its retention in the ER, light and heavy chains of the HBsAg mAbs were expressed including a C-terminal DDEL signal fusion [18]. One C-terminal peptide carrying DDEL was detected in the LC-ESI MS and MS/MS profiles of the heavy chain from the ER-retained mAb (Fig 2A). This peptide resulted from a trypsin miss cleavage after the C-terminal K_467_ (S_460_LSLSPGK_467_DDEL_471_). Moreover, the C-terminal G_234_ECDDEL_240_ sequence was identified in the tryptic digest of the light chain (Fig 2B), thus confirming the presence of the DDEL retention signals on both light and heavy chains of the ER-retained mAb. These results demonstrate that there is no C-terminal proteolysis occurring on the recombinant proteins within the diatom cells.

**Fig 3:**
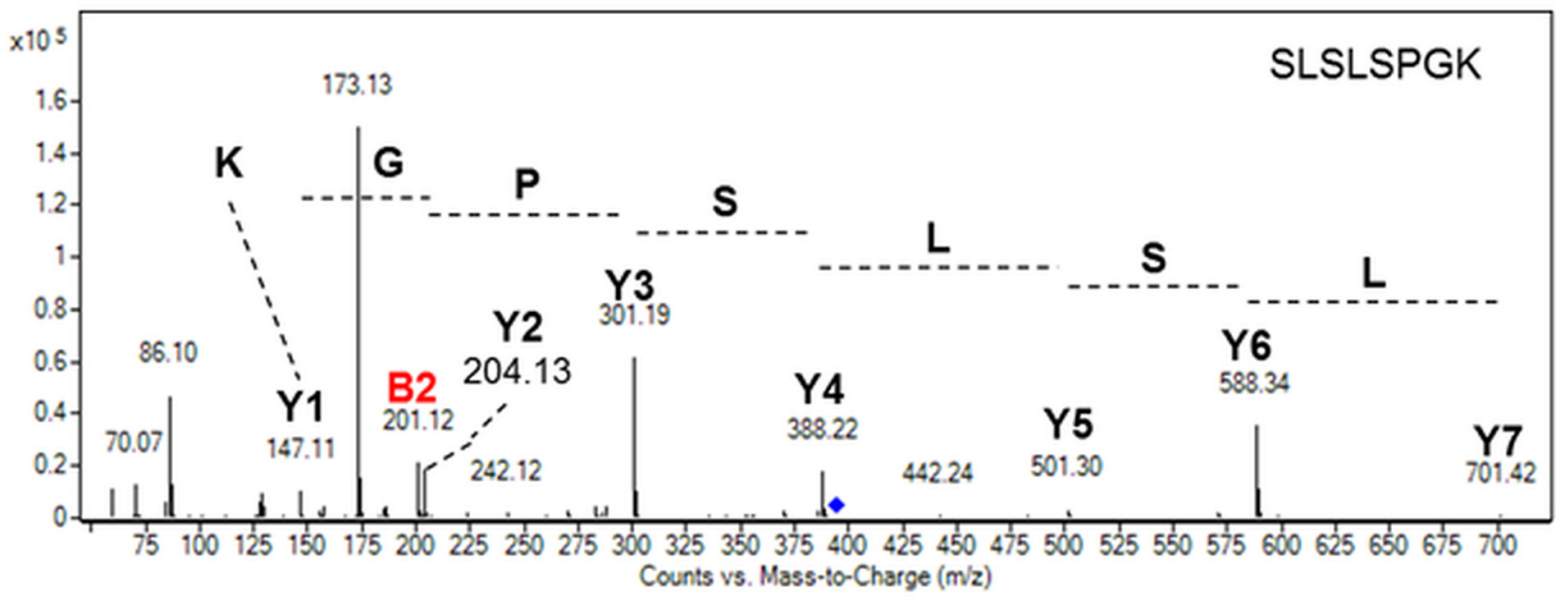
Mass spectrometry analysis of the C-terminal peptide of the heavy chain of the secreted mAb produced in *P*. *tricornutum*. MS/MS spectrum of the S_460_LSLSPGK_467_ ([M+2H]^2+^ precursor ion at *m/z* 394,73). Y and B ions have been identified and are reported in the spectrum. The peptide can be rebuilt from either ones.

Additionally, particular attention has been paid to the C-terminal sequence of the secreted mAb as it is well established that C-terminal lysine processing of the heavy chain is one common modification occurring frequently on recombinant mAb [3,20,21]. Indeed, the terminal lysine residues from the C-terminal sequence PGK of the heavy chain are susceptible to removal by endogenous carboxypeptidases [21]. This is known to occur during the production cell culture process and typically results in the production of heterogeneous mAbs [22,23]. The C-terminal peptide (S_460_LSLSPGK_467_) of the heavy chain from the secreted mAb produced in *P*. *tricornutum* has been analyzed by MS and MS/MS. The analysis of the different fragment ions observed in the MS/MS spectrum confirmed the presence of the C-terminal lysine (Fig 3), thus demonstrating the capability of *P*. *tricornutum* to produce recombinant antibodies with consistent and homogeneous C-terminal ends in contrast to secreted mAbs from CHO cells which are highly heterogeneous.

### N-terminal signal peptide cleavage

**Fig 4:**
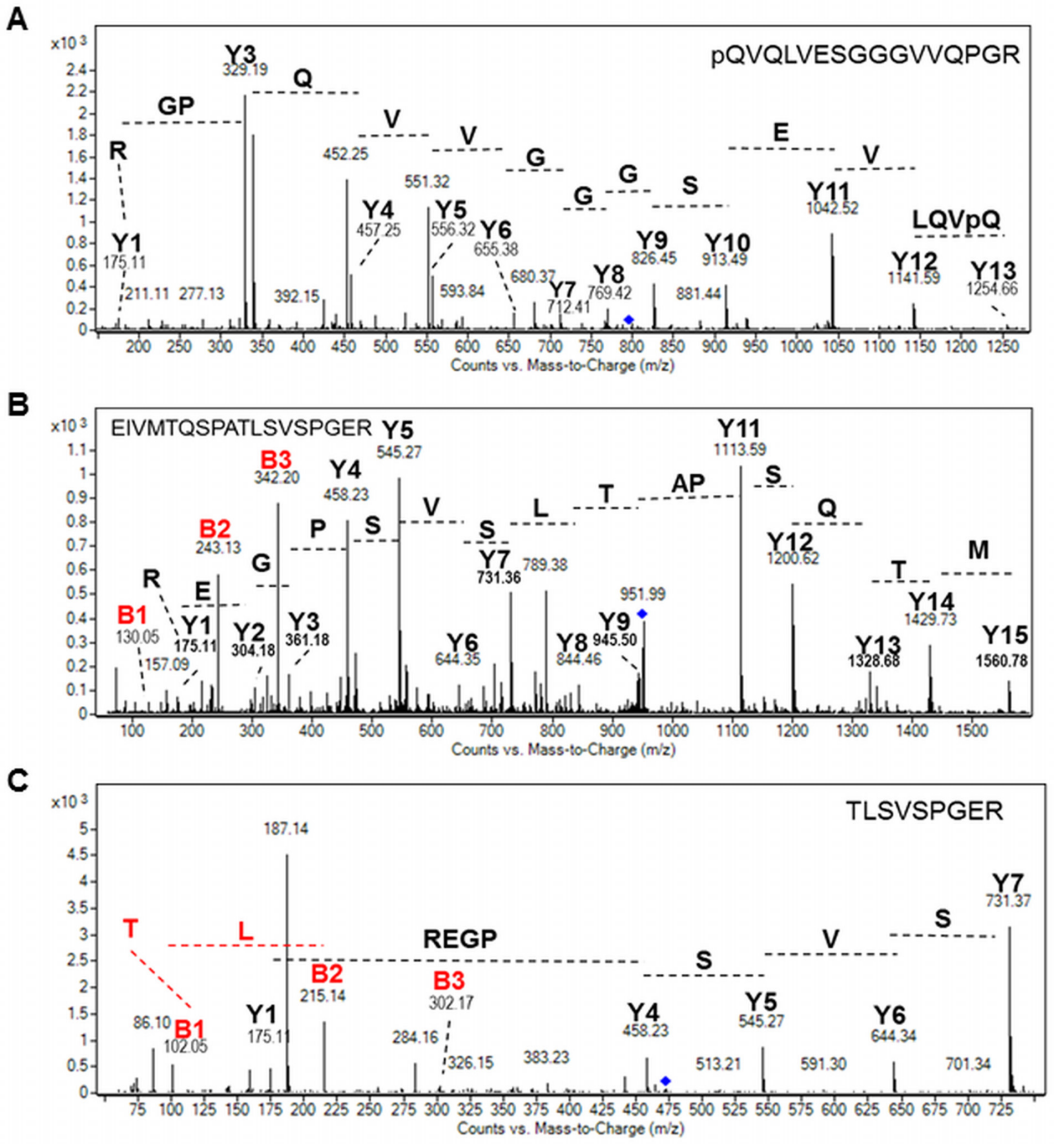
Analysis of the N-terminal peptides of the secreted and ER-retained mAbs by mass spectrometry. A) MS/MS spectrum of the pQ_20_VQLVESGGGVVQPGRSLR_38_ peptide from the heavy chain ([M+2H]^2+^ precursor ion at *m/z* 796.92). B) MS/MS spectrum of the E_23_IVMTQSPATLSVSPGER_40_ from the light chain ([M+2H]^2+^ precursor ion at *m/z* 951.48). C) MS/MS spectrum of the TLSVSPGER from the light chain (M+2H]^2+^ precursor ion at *m/z* 473.25). pQ is indicated a N-terminal pyroglutamine residue formed by cyclisation of glucosamine. For all spectra, Y and B ions have been identified and allowed peptide sequencing.

Signal peptides play a crucial role in the targeting and translocation of secreted proteins within the lumen of the ER in eukaryotes. This often represents a limiting factor for the production of recombinant mAbs in eukaryotic cells. When the nascent protein with its signal peptide emerges from the ribosome, it is recognized by the signal recognition particle (SRP) and form a ribosome-signal peptide-SRP complex which is directing the nascent protein to the ER membrane. Such complex is then interacting with the SRP receptor, allowing the ribosome to bond to the translocon and finally leading to the transfer of the polypeptide into the ER. The SRP if then released and finally the signal peptide is recognized and cleaved off by signal peptide peptidases [24,25]. Genes encoding for putative signal peptide peptidases have been predicted and annotated in the *P*. *tricornutum* genome [26] (http://genome.jgi-psf.org/Phatr2/Phatr2.home.html). However, the ability of these ER peptidases to cleave signal peptides of recombinant proteins in this diatom has not yet been investigated. Therefore, particular attention has been paid to the N-terminal sequences of the heavy and light chains of the algae-made antibodies. To determine the signal peptide cleavage sites occurring within the diatom cells for both light and heavy chains, manual searches were done among all of the MS and MS/MS data. In agreement with prediction and as observed in mammalian cells [27], cleavage of heavy chain signal peptide occurred after C_19_ (M_1_EFGLSWVFLVALLRGVQC_19_) in both secreted and ER-retained recombinant antibodies (Figs 1C and 4A), thus demonstrating that the microalga is able to efficiently cleave the signal peptide of the heavy chain using a signal peptide peptidase mechanism that is similar to the one occurring in mammalian cells and other eukaryotes [27–29]. This completes and reinforces previous datasets which conclude that the diatom *P*. *tricornutum* possesses the ER machinery required for polypeptide translocation and protein quality control [8,11]. Such mechanisms are similar if not identical to the ones previously described for other eukaryotes [30]. It is worth noting that the N-terminal peptide identified by MS and MS/MS for the heavy chain carries a N-terminal pyroglutamine residue (noted pQ on the Fig 4A). This modification is formed by cyclisation of the glutamine residue (Q) and is very often reported for recombinant mAbs produced in mammalian cells [3,20,21]. Such modification is described to be a chemical non-enzymatic modification occurring for the majority (up to 90%) during the cellular process [31,32]. It is also present on human IgGs as most of the N-terminal glutamine of heavy chains of human IgGs is cyclized to pyroglutamate [33].

Concerning the light chains, the N-terminal peptides resulting from cleavage of the light chain signal peptide after the G_22_ (E_23_IVMTQSPATLSVSPGER_40_) were detected in both ER-retained and secreted recombinant mAbs (Figs 1D and 4B). This result was expected as the signal peptide is known to be 22 amino acids long for the light chain of human antibodies [27], meaning again that the microalga cell is able to cleave the signal peptide from the light chain following a process similar to those observed in mammalian cells and other eukaryotes. However, a second N-terminal peptide resulting from a cleavage after the A_31_ (T_32_LSVSPGER_40_) was also identified by LC-ESI MS and MS/MS analysis of the light chain N-terminal peptides (Figs 1D and 4C). Comparison of the ion intensities for both light chain N-terminal peptides showed that the second N-terminal peptide resulting from the cleavage after the A_31_ accounts for around 90% of the total. Since this unexpected N-terminal peptide is observed in both secreted and retained mAbs, we postulate that it may result from the recognition of a second cleavage site by the diatom signal peptide peptidases, rather than post-synthesis proteolytic degradation of the light chains.

### Other post-translational modifications

**Fig 5:**
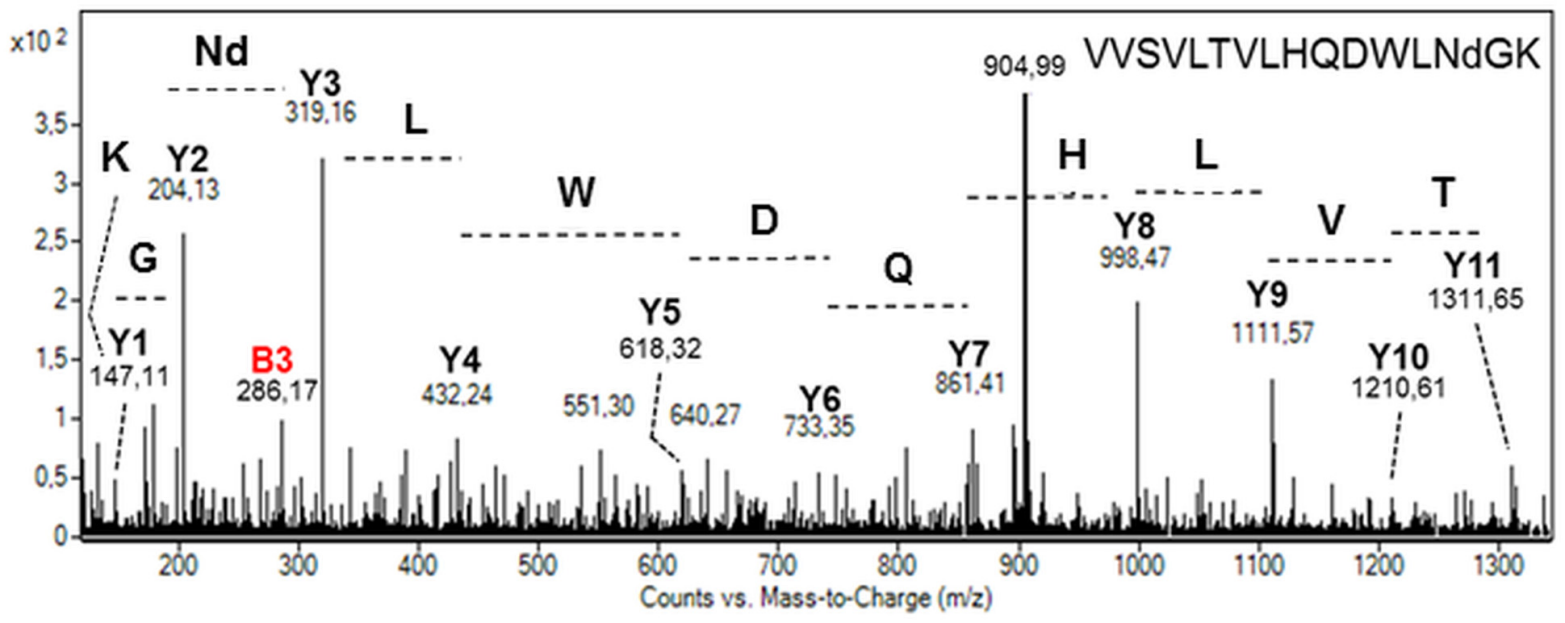
MS/MS spectrum of the peptide V_322_VSVLTVLHQDWLNGK_337_ from the heavy chain ([M+2H]^2+^ precursor ion at *m/z* 904.99) showing the deamidation carried out by the specific N_335_. The asparagine deamidation is indicated by Nd both on the spectrum and on the peptide amino acid sequence. Y and B ions have been identified and are reported in the spectrum. The peptide can be rebuilt from either ones.

Search for other post-translational modifications has been performed on both ER-retained and secreted mAbs. Whereas no oxidation of methionine residues has been observed, one deamidation of the specific N_335_ residue on the peptide V_322_VSVLTVLHQDWLNGK_337_ has been observed by MS and further confirmed by MS/MS (Fig 5). Such asparagine deamidation has already been described on mAbs [34] and is influenced by many environmental factors such as pH, temperature, buffer composition [35,36].

### *N*-glycosylation of algae-made mAbs

**Fig 6:**
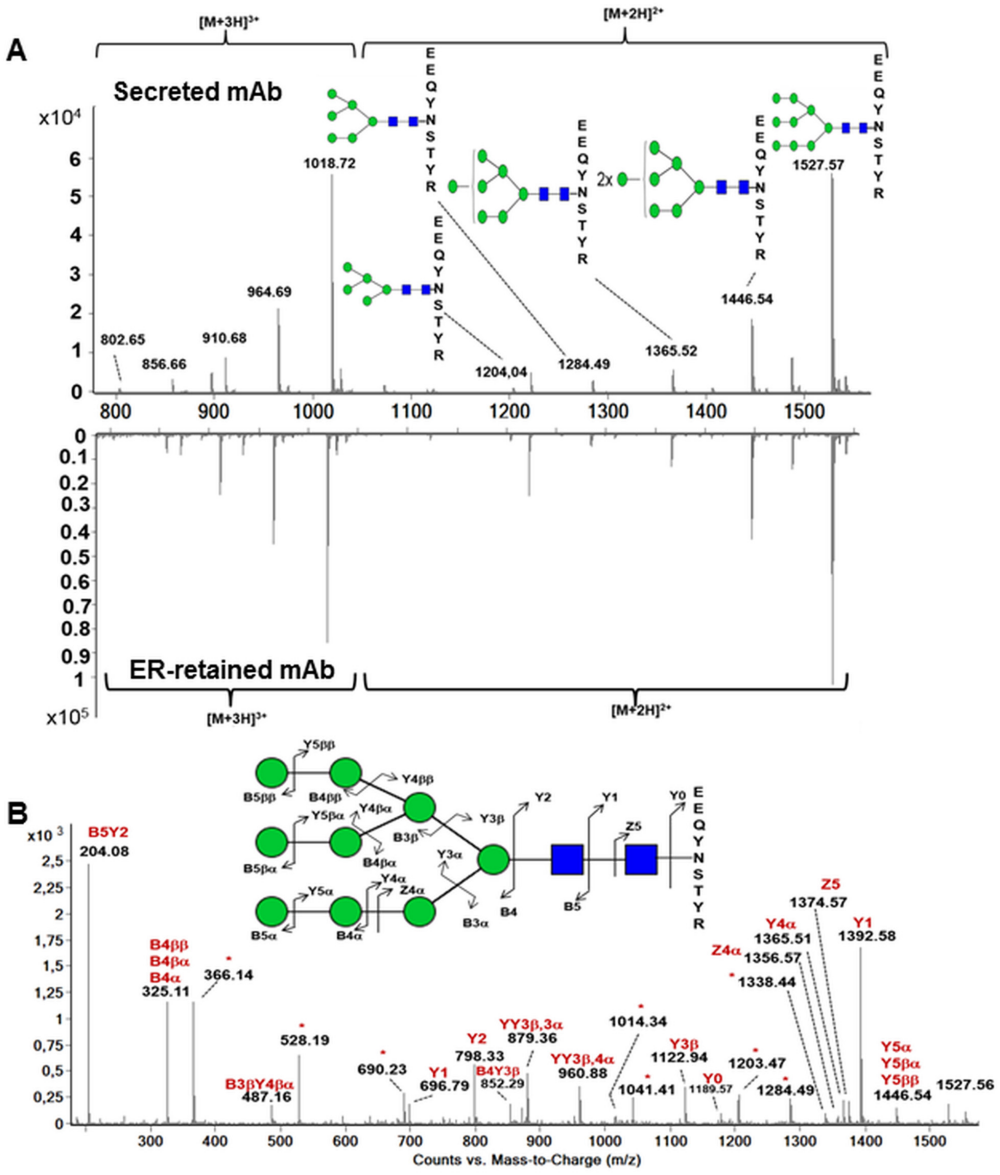
Analysis of the *N-*glycosylation of the heavy chain by mass spectrometry. **A)** MS spectrum of the glycopeptide from the heavy chain of the secreted and ER-retained antibodies produced in *P*. *tricornutum*. **B)** MS/MS spectrum of the glycopeptide bearing a Man–9 structure ([M+2H]^2+^ precursor ion at *m/z* 1527.57). Fragment ions were annotated using Domon and Costello nomenclature [37]. The asterisks indicate multiple fragment ions that have been identified but not annotated on the spectrum. Glycan structures are annotated with their proposed carbohydrate structure according to the symbolic nomenclature from the Consortium for Functional Glycomics [38]. Black square: *N-*acetylglucosamine; green circle: mannose.

Then, we focused on the *N*-glycosylation of the algae-made mAbs as it is well known that *N-*glycosylation is an important quality attribute influencing functionality and stability of such recombinant glycoproteins [12]. Firstly, *N-*glycan profiles present on the ER-retained and secreted mAbs were investigated. A search for glycopeptides in LC-ESI MS analysis was carried out using diagnostic ions at m/z 204 and 366. These fragments correspond to the oxonium ions of *N*-acetylhexosamine (HexNAc) residues and of hexose-HexNAc disaccharides observed in the MS/MS spectra of glycopeptides, respectively. In both ER-retained and secreted heavy chains, a single glycopeptide with various glycoforms was detected (Fig 6A). For the ER-retained antibody, the glycopeptide MS profile indicated that the heavy chain is glycosylated by high mannose-type *N*-glycans ranging from Man–5 to Man–9, with the major structures being Man–8 and Man–9 (Fig 6A). Structures of these glycopeptides were confirmed by analysis of fragmentation patterns obtained by MS/MS as illustrated for Man-9-containing E_313_EQYNSTYR_321_ glycopeptide in Fig 6B and for other glycoforms. In addition to diagnostic ions at m/z 204 and 366, m/z ions corresponding to the peptide carrying one GlcNAc (Y1 fragment ion) up to Man_5_GlcNAc_2_ (Y5 fragment ions) or larger oligomannosides were also detected in the MS/MS spectra. The same analysis was carried out on glycopeptides from the secreted mAb. Surprisingly, similar glycoforms were detected on the N_317_ of the heavy chain from the secreted mAb. As shown in Fig 6A, only minor differences in the relative percentage from one glycan to another can be observed when comparing the ER-retained and secreted mAb *N*-glycan profiles.

**Fig 7:**
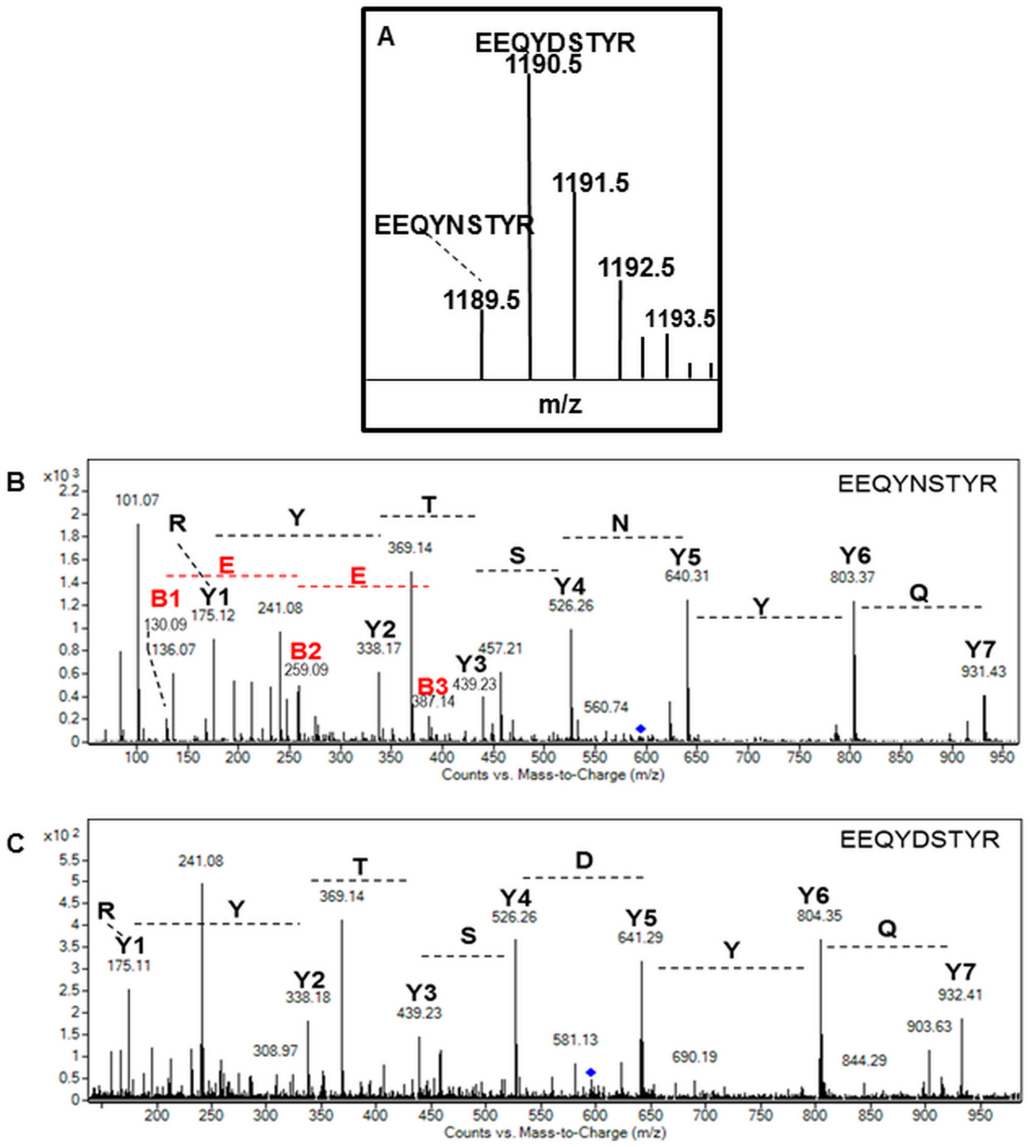
*N-*glycosylation site occupancy by mass spectrometry after PNGase F digestion. **A)** MS spectrum of the PNGase F deglycosylated peptide E_313_EQYDSTYR_321_ ([M+2H]^2+^ precursor ion at *m/z* 595.76). **B)** MS/MS spectrum confirming the identification of the non-glycosylated E_313_EQYNSTYR_321_ peptide ([M+2H]^2+^ precursor ion at *m/z* 595.27) **C)** MS/MS spectrum of the PNGase F deglycosylated E_313_EQYDSTYR_321_ peptide ([M+2H]^2+^ precursor ion at *m/z* 595.76) from the heavy chain of the algae-made antibodies. For all spectra, Y and B ions have been identified and allowed peptide sequencing.

We also focused on the N-glycosylation site occupancy located on the N_317_ on the primary protein sequence of the HBsAg mAbs produced in *P*. *tricornutum* presents a unique potential *N-*glycosylation site located in the C_H_2 domain of the heavy chain (Fig 1C). However, the heavy chain was revealed as two bands after NuPAGE gel electrophoresis separation, suggesting partial post-translational modifications of the heavy chains for both ER-retained and secreted mAbs (Fig 1B). To investigate whether the two heavy chain bands were due to partial *N-*glycosylation, LC-ESI MS analyses of tryptic digested heavy chains were carried out before and after deglycosylation with the peptide *N*-glycosidase F (PNGase F; [39,40]). Indeed, this enzyme is able to cleave off glycans *N*-linked to proteins and, at the same time, to convert asparagine (N) residues of the occupied *N*-glycosylation site into aspartic acid (D). This shift in peptide mass (+1 Da) enables the identification of glycosylated peptides by mass spectrometry [41]. As the recombinant mAbs were found to carry out high mannose-type *N-*glycans, the PNGase F was chosen for the mAb deglycosylation experiments instead of PNGase A, which is required specifically for glycoproteins carrying α(1, 3)-fucose residues on the proximal glucosamine of *N-*glycans [42]. Before deglycosylation, non-glycosylated E_313_EQYNSTYR_321_ peptides were detected at m/z 1189.5 in the heavy chain peptide digests of ER-retained and secreted mAbs. After deglycosylation, additional aspartic acid-containing E_313_EQYDSTYR_321_ peptides at m/z 1190.5 were found (Fig 7A and 7C). Amino acid sequences of these two peptides were confirmed by LC-ESI MS/MS analysis (Fig 7B and 7C). The ratio between both peptides using single and dicharged ion intensities indicated that around 70% of the N_317_ is glycosylated in both antibodies (Table 1). These results have been confirmed by densitometry analysis performed on the Coomassie blue stained gel electrophoresis (Fig 1A and Table 1). Such partial *N-*glycosylation has already been described for mAbs produced in CHO cells which can present, in the worst case scenario, up to 52% of non-glycosylated mAbs depending of the culture conditions [43]. Therefore, the diatom is as efficient as other expression systems, as far as mAb *N-*glycan site occupancy (macroheterogeneity) is concerned.

**Table 1:** Quantification of the *N-*glycosylation site occupancy.

	Secreted mAb		ER-retained mAb	
Glycosylation site occupancy	% of glycosylation based on ion intensities	% of glycosylation based on densitometry	% of glycosylation based on ion intensities	% of glycosylation based on densitometry
EEQYDSTYR	77	80	70	68
EEQYNSTYR	23	20	30	32

The *N-*glycosylation site occupancy was calculated from the comparison of the ion intensity of the non-glycosylated versus deglycosylated peptides using single and discharged specific ions and from densitometry analysis of the two bands corresponding to heavy chains observed on the NuPAGE gel.

In eukaryotes, the *N-*glycan oligosaccharide precursor Glc_3_Man_9_GlcNAc_2_ is first synthesized and then transferred *“en bloc”* onto the asparagine of the consensus *N*-glycosylation sites (NXS/T/C) of nascent proteins. This initial step of the *N*-glycosylation processing occurs within the ER. This glycan precursor is submitted to the elimination of the terminal glucose units catalyzed by the α-glucosidases I and II followed by removal of a single mannose residue by an ER-mannosidase. This ER maturation allows the interaction with ER-resident lectins that ensure the quality control of secreted proteins. As a consequence, detection of Man–8 and Man–9 on the DDEL ER-retained mAbs is consistent with its retention in the ER. The presence of Man–5 to Man–7, in addition to the expected Man–8 and Man–9 structures, suggests that the ER retained algae-made antibody has been partially trimmed by the α-mannosidase I, an enzyme located in the *cis*-Golgi in eukaryotes and able to sequentially convert Man–8 into Man–5 by elimination of terminal α(1, 2)-mannose residues. Although the location in diatoms of such mannosidases has not been elucidated to date, this observation is consistent with the model of ER retention, which is in fact based on retrograde trafficking from *cis*-Golgi to the ER. Indeed in this model, ER retrieval occurs by recognition of the protein C-terminal retention signal (DDEL in this study) by a specific receptor in the *cis*-Golgi network, followed by retrograde transport back to the ER. Such a *N-*glycosylation profile was previously reported for other ER-retained antibodies that have been earlier produced in transgenic plants using either a HDEL or KDEL retention signal [44–49].

The *N*-glycosylation bore by the heavy chain of the secreted mAb is more questionable, since no major differences in the *N-*glycan profiles were observed as compared to the *N-*glycan profile of the ER-retained mAbs. It is worth noting that glycans *N*-linked to *P*. *tricornutum* endogenous glycoproteins are mainly Man–5 to Man–9 high mannose-type *N*-glycans with Man–5 and Man–9 being of equal importance as previously described [8]. Different profiles have been observed in the secreted mAbs, as Man–9 and Man–8 were the most important glycoforms, with Man–7 to Man–5 being of minor importance. Such discrepancy may be due to the fact that these glycosylation analyses have been performed on an entire population of endogenous glycoproteins [8], whereas, in the present study, the glycosylation analysis was performed on a single individual secreted glycoprotein. Moreover, the conformation of the Fc part may explain the lower maturation of the *N*-glycans that have been found in the secreted algae-made antibody. Indeed, the two constitutive oligosaccharides of IgGs are buried in the interstitial region between the two C_H_2 domains of the Fc fragments [50–52]. As a consequence, these oligosaccharides are known to be less accessible to Golgi processing glycosyltransferases, which could result in an incomplete maturation of the *N*-linked glycans during secretion [53]. This could explain the absence of complex type *N*-glycans on the secreted anti-HBsAg algae-made antibody. It would be necessary in future experiments to express and evaluate the glycosylation profiles of other recombinant mAbs and other secreted glycoproteins within *P*. *tricornutum*.

One *N*-acetylglucosaminyltransferase I (GnT I) gene was previously predicted in the *P*. *tricornutum* genome and was demonstrated to be functional and able to convert Man–5 into complex-type *N*-glycans. Indeed, this GnT I from *P*. *tricornutum* has been proven to be active *in vivo* by complementation of the maturation of complex-type *N*-glycans in the CHO *Lec1* mutant [8]. In contrast, only traces of such complex *N*-glycans were detected in the *N-*glycan profile isolated from the total *P*. *tricornutum* protein extract [8]. In this work, a search for specific diagnostic ions did not allow any detection of complex-type *N*-glycans, including fucosylated forms, on the secreted mAbs produced in *P*. *tricornutum*. These are probably either absent or at too low abundance for detection. As a consequence, Golgi maturation of high mannose-type *N-*glycans seems to be inefficient in this diatom or restricted to a certain population of secreted proteins. In this context, it will be interesting to better understand the regulation of the GnT I activity within *P*. *tricornutum* and to evaluate whether, in this microalgae, culture conditions and cell physiology may influence its glycosylation capabilities and allow the formation of complex-type *N-*glycans. Such effects of the culture and growth conditions on the *N-*glycosylation profiles of recombinant mAbs have already been extensively described for production in transgenic plant and mammalian cells [43,54–60].

The two recombinant anti-HBsAg mAbs that we analyzed in this paper and that carry high mannose-type *N-*glycans were previously described to be functional *in vitro*, as they are able to recognize and bind to their specific antigen [18,19]. This is not surprising as the antigen binding functions are independent of the *N-*glycosylation of the Fc part. This would probably be untrue for the effector functions of these mAbs, including activation of the complement cascade by binding to C1q. Indeed, it has been well established that complex-type *N*-glycans, especially biantennary *N*-glycans bearing β(1, 4)-galactose in the terminal position are required for optimal binding of antibodies to all classes of receptors [51,61,62]. The presence of different glycans on the Fc part, such as high mannose-type glycans that are *N*-linked to the algae-made antibodies, might alter the effector functions of the anti-HBsAg mAbs by provoking distortion of the conformation of the Fc domains [50–52], thus leading to a reduction in its affinity for Fcγ receptors and C1q, as well as inducing a deficiency in antibody-dependent cell-mediated cytotoxicity (ADCC) [63]. Furthermore, *in vivo* clearance of antibodies with high mannose-type *N*-glycans is increased by binding to the macrophage mannose receptor in the liver [64–66]. Therefore, taking into account the current *N*-glycan profiles of the ER-retained and secreted anti-HBsAg mAbs, we conclude that production in *P*. *tricornutum* of mAbs that are suitable for human injectable therapy, thus necessitating mAb effector functions, will require in the future intensive understanding of the diatom endogenous glycosylation machinery. Moreover, extensive glyco-engineering efforts have to be undertaken for optimizing mAbs production in this diatom.

Taken together, the data presented here highlight the potential use of *P*. *tricornutum* as a suitable platform for producing *N-*glycoproteins carrying high mannose-type *N*-glycans. This capability to produce and add mannose terminating *N-*glycans onto recombinant proteins could represent an eminent advantage for the production of glycosylated biopharmaceuticals, such as glucocerebrosidase (GBA), which requires effective targeting and internalisation into macrophages through the recognition of terminal mannose residues on the *N-*glycans by macrophage cell surface mannose receptors. Glucocerebrosidase is a lysosomal enzyme used in enzyme replacement therapy for treating Gaucher’s disease. Exogenous glucocerebrosidase is administered intravenously into patients and glucocerebrosidase preparation currently involves either extraction directly from human placenta or recombinant expression in CHO cells (Cerezyme^®^), which requires *in vitro* post-purification exoglycosidase digestions to unravel the trimannose core (Man_3_GlcNAc_2_) using a combination of sialidase, galactosidase, and *N-*acetylglucosaminidase. These *in vitro* glycoengineering steps increase considerably the production costs [67]. Therefore, alternative expression systems that are capable of producing mannose terminated *N-*glycans have been developed as safe and cost effective production methods. These include cultured carrot cells [68] or transgenic plants [69]. The results described in this paper suggest that *P*. *tricornutum* could, in the near future, become an appropriate platform for the production of a glucocerebrosidase (GBA) biobetter that would be directly carry high mannose-type *N-*glycan structures without *in vitro* trimming.

## Conclusions

The data presented here show that the diatom *P*. *tricornutum* could be used for producing recombinant *N-*glycoproteins carrying high mannose-type *N*-glycans. Moreover, detailed analysis of the C-terminal ends of the heavy and light chains demonstrated the capability of *P*. *tricornutum* to produce recombinant antibodies with consistent and homogeneous C-terminal ends without proteolysis. Additionally, the MS and MS/MS analyses of the heavy chain *N*-terminal peptide in both secreted and ER-retained recombinant antibodies demonstrated that this diatom is able to efficiently cleave the signal peptide using a signal peptide peptidase mechanism that is similar to the one occurring in mammalian cells and other eukaryotes.
